# Comparing the Impact of COVID-19-Related Social Distancing on Mood and Psychiatric Indicators in Sexual and Gender Minority (SGM) and Non-SGM Individuals

**DOI:** 10.3389/fpsyt.2020.590318

**Published:** 2020-12-22

**Authors:** Craig Rodriguez-Seijas, Eric C. Fields, Ryan Bottary, Sarah M. Kark, Michael R. Goldstein, Elizabeth A. Kensinger, Jessica D. Payne, Tony J. Cunningham

**Affiliations:** ^1^Department of Psychology, University of Michigan, Ann Arbor, MI, United States; ^2^Department of Psychology, Brandeis University, Waltham, MA, United States; ^3^Department of Psychology and Neuroscience, Boston College, Chestnut Hill, MA, United States; ^4^Division of Sleep Medicine, Harvard Medical School, Boston, MA, United States; ^5^Department of Neurobiology and Behavior, Center for the Neurobiology of Learning and Memory, University of California, Irvine, Irvine, CA, United States; ^6^Department of Neurology at Harvard Medical School, BIDMC, Boston, MA, United States; ^7^Department of Psychology, University of Notre Dame, Notre Dame, IN, United States; ^8^Department of Psychiatry, Harvard Medical School, Boston, MA, United States; ^9^Department of Psychiatry, Beth Israel Deaconess Medical Center, Boston, MA, United States

**Keywords:** COVID-19, gender, sexual orientation, well-being, depression, social isolation, mood

## Abstract

Empirical evidence demonstrates mental health disparities between sexual and gender minority individuals (SGM) compared with cisgender heterosexual individuals. SGM individuals report elevated rates of emotional distress, symptoms related to mood and anxiety disorders, self-harm, and suicidal ideation and behavior. Social support is inversely related to psychiatric symptoms, regardless of SGM status. The COVID-19 pandemic—with its associated limited social interactions—represents an unprecedented period of acute distress with potential reductions in accessibility of social support, which might be of particular concern for SGM individuals' mental well-being. In the present study, we explored the extent to which potential changes in mental health outcomes (depressive symptoms, worry, perceived stress, positive and negative affect) throughout the duration of the pandemic were related to differences in perceptions of social support and engagement in virtual social activity, as a function of SGM status. Utilizing a large sample of US adults (*N* = 1,014; 18% reported SGM status), we assessed psychiatric symptoms, perceptions of social isolation, and amount of time spent socializing virtually at 3 time windows during the pandemic (between March 21 and May 21). Although SGM individuals reported greater levels of depression compared with non-SGM individuals at all 3 time points, there was no interaction between time and SGM status. Across all participants, mental health outcomes improved across time. Perceived social isolation was associated with poorer mental health outcomes. Further, time spent engaging in virtual socialization was associated with reduced depression, but only for those in self-reported quarantine. We discuss these results in terms of the nature of our sample and its impact on the generalizability of these findings to other SGM samples as well as directions for future research aimed at understanding potential health disparities in the face of the COVID-19 pandemic.

## Introduction

The Coronavirus Disease 2019 (COVID-19) pandemic is unprecedented in most of our lifetimes and has had far reaching effects worldwide. At the time of this publication, we are only beginning to grasp the full extent of this international public health crisis. With this unique time in human history comes the realization that we have little understanding of the differential impact of large-scale public health measures being implemented. While there has necessarily been focus on the physical health implications of the pandemic, it is becoming increasingly clear that there are important mental health repercussions that are only beginning to be understood (1). Worse mental health outcomes have been reported as a direct function of COVID-19 infection [e.g., (2)] as well as due to indirect distress related to the pandemic [e.g. (3, 4)]. Apart from the general distress related to the COVID-19 virus itself and potential morbidity, psychosocial disruptions and alienation resultant from measures designed to contain the spread of the disease hold the potential to further compromise mental health through curtailed opportunities to engage in social activities. Further, these deleterious effects might not universally affect all persons in equal magnitude.

Sexual and gender minority (SGM) individuals—those reporting sexual orientation and gender identity other than heterosexual and cisgender—represent one population likely to be disproportionately impacted by COVID-19 and public health responses to the disease. SGM individuals typically experience higher rates of poverty (5), housing instability (6), food insecurity (7), lack of healthcare insurance (8), and employment within industries negatively affected by and with higher infection potential (9) compared with their cisgender heterosexual counterparts. Independent of the COVID-19 pandemic, a robust literature documents psychosocial health disparities between SGM and cisgender heterosexual individuals. When compared with cisgender heterosexual individuals, SGM persons demonstrate higher prevalence of mood and anxiety disorders, suicidal ideation and behavior, as well as problematic substance use (10–19). Further, psychosocial health disparities observed among SGM populations exert a synergistic effect in the ways in which they compromise SGM well-being (20, 21). These health disparities are largely driven by minority stress processes (22–24); sexual and gender minority-based stressors operate in direct and indirect ways to compromise well-being (25, 26). Stigma and discrimination against SGM individuals, especially for those with intersecting marginalized identities, have contributed to barriers in accessing healthcare, employment, and other socioeconomic resources. The global public health response to the COVID-19 pandemic might potentiate psychosocial threats to mental health among SGM individuals (8).

Social support promotes well-being. Conversely, social isolation compromises health (27). Perceived social support attenuates the impact of stressful life events on psychological distress (28–31) and is negatively associated with depressive symptoms specifically (32–35), as well as psychiatric distress more generally (6). Empirical evidence further documents the importance of social support for SGM individuals' mental health. Perceptions of support from family and friends are negatively associated with mental health (36–39) outcomes. Social support not only directly impacts mental health, but also indirectly through engagement in effective behavioral coping mechanisms (40, 41).

However, SGM individuals experience greater social isolation and less social support than their cisgender heterosexual peers (42, 43). The importance of social support for SGM individuals' health is reflected in an explicit focus on facilitating supportive relationships in evidence-based treatment of SGM individuals' psychosocial health (44–46). Emerging literature further highlights the importance of social support for navigating COVID-19-related distress. For example, in one study (3), adults in Egypt reported seeking increased support from friends and family members in response to the pandemic. Additionally, data from Italian adults in high- and low-contagion regions demonstrates the buffering effects of both in-person and virtual social support on psychiatric distress symptoms (47). Facing increased social exclusion and marginalization at a population level alongside worse group-based mental health outcomes, resultant from minority stress, when compared with cisgender heterosexual individuals, it is possible that social restrictions aimed at containing the spread of COVID-19 might disproportionately compromise the mental health of SGM individuals.

The goal of the current study was to compare the impact of (1) nationwide business closures and stay-at-home orders at the onset of the United States response to the COVID-19 pandemic, (2) perceptions of social isolation and time spent engaging in virtual socializing activities, and (3) the interrelatedness of these variables on ratings of mood, depressive symptoms, worry, and perceived stress between SGM and cisgender heterosexual individuals. As preregistered at https://osf.io/kg6bu, we hypothesized that SGM individuals will report increased symptoms of psychiatric distress at the start of the assessment period when compared with cisgender heterosexual individuals. Further, we predict that the disparity would become exacerbated over the course of the assessment period. We also hypothesize that perceptions of social isolation will be positively associated with, and reports of time spent socializing virtually will be negatively associated with, mental health outcomes across time, and that this relationship will be stronger for SGM individuals (i.e., social support will be more impactful for SGM persons than cisgender heterosexual individuals).

## Methods

### Participants

As part of a larger, ongoing study exploring the mental health repercussions of the COVID-19 pandemic and response measures, online recruitment for this report began on March 20, 2020. During the course of the recruitment period, *N* = 1,930 participants completed the online informed consent and were enrolled in the study. Of this initial recruitment, *N* = 1,462 completed the initial demographic survey, which was required before daily surveys began. Of this total sample, *n* = 1,171 reported cis-gender identity and heterosexual sexual orientation and *n* = 291 reported SGM status (19.9% of total sample). As the time course of the spread and response measures to COVID-19 differed by country, here we only included participants in our study from the United States (cisgender heterosexual *n* = 833; SGM *n* = 181) to minimize the variability in timelines. All English-speaking adults 18+ from anywhere in the world were eligible for the study, regardless of pre-existing mental health or medical conditions. Only study personnel were ineligible for participation. Recruitment relied primarily on contact with previous participants, dissemination through professional networks, social media, and word of mouth. The age of participants in this sample ranged from 18 to 90 years old (M = 36.7, SD = 16.0). See Table 1 for additional demographics. Compensation for participation was in the form of raffle entries for gift cards. The Boston College Institutional Review Board approved all consent and assessment procedures.

**Table 1:** Demographics of SGM and non-SGM samples.

	**ALL**	**Non-SGM**	**SGM**
***N***	**1,014**	**833**	**181**
**Age**			
Mean	36.65	37.96	30.62
Standard deviation	15.96	16.54	11.19
Minimum	18	18	18
1st Quarilte	26	26	23
Median	31	31	28
3rd Quartile	42.75	46	34
Max	90	90	83
**Ethnicity**			
Hispanic	6%	6%	5%
Not Hispanic	93%	93%	93%
Prefer not to say	1%	1%	1%
**Race**			
African American	2%	0%	3%
Asian	8%	9%	8%
White	81%	81%	81%
Hispanic/Latinx	2%	3%	2%
Native Hawaiian or Other Pacific Islander	0%	0%	0%
American Indian/Alaska Native	0%	1%	0%
More than one race/Prefer to self-describe	6%	6%	6%
Unknown	0%	0%	0%
Prefer not to say	0%	0%	0%
**Gender**			
Female	81%	82%	76%
Male	18%	18%	16%
Non-binary/third gender	1%	0%	7%
Other	0%	0%	1%
**Biological Sex**			
Female	82%	81%	85%
Male	18%	19%	15%
**Gender Identity**			
Cisgender	99%	1.00%	94%
Transgender	1%	0%	4%
Unknown	0%	0%	2%
**Sexual Orientation**			
Straight	82%	100%	1%
Bisexual	12%	0%	70%
Gay	3%	0%	19%
Other	2%	0%	11%
**Education**			
Some high school	0%	0%	1%
High school diploma/GED	2%	2%	3%
Some college	14%	13%	19%
Bachelor's degree	28%	29%	25%
Some post-graduate	11%	11%	11%
Post-graduate or professional degree	45%	45%	41%
**Marital Status**			
Single	33%	31%	43%
In a relationship	26%	25%	31%
Married	34%	36%	24%
Divorced/separated	5%	5%	3%
Widowed	2%	3%	0%
**Serious Medical Problems**			
No	92%	93%	90%
Yes	8%	7%	10%
**Income**			
$0–25,000	8%	7%	12%
$25,001–50,000	14%	12%	23%
$50,001–75,000	18%	18%	18%
$75,001–100,000	16%	17%	14%
$100,001–150,000	20%	20%	15%
$150,001,−250,000	15%	16%	12%
$250,000+	9%	10%	7%
**Student**			
No	76%	78%	69%
Yes	24%	22%	31%
**Employed**			
Yes	79%	79%	80%
No	21%	21%	20%

*All age metrics reported are in “years.” All other measures are reported as the percentages of each group (All, SGM, non-SGM) that identify as each demographic variable*.

### Assessment Materials

#### Demographic Survey

Immediately after consenting, participants were sent an initial demographic survey. Completion of this survey was required before receiving any further assessments. Participants self-reported natal sex, current gender identity, sexual orientation, race/ethnicity, age, socioeconomic status, marital status, military status, education level, number of dependents, and whether or not they had ever received a previous diagnosis of a serious mental health and medical condition. Congruent with the previous literature documenting higher psychiatric disorder prevalence among SGM compared with non-SGM populations, 51% of the SGM sample reported being previously diagnosed with a serious mental health condition compared with 26% of the non-SGM sample. However, there were high rates of missingness in the dichotomous previous mental health diagnosis variable. Fifty-two percentage of SGM and 49% of non-SGM persons did not provide information on previous mental health disorder diagnoses. The demographic survey questions used for all participants can be found here: https://bit.ly/BC-DEMOS.

#### Daily Survey

After completion of the Demographic Survey, participants were immediately enrolled to begin receiving daily assessment surveys. Two versions of our daily survey were utilized throughout the duration of the assessment period: a Short Version and a Full Version. The Short Version was created to reduce participant burden during the longitudinal study design. Relevant to this report, the Short Survey included questions of subjective experience of stress, time spent virtually socializing, and perception of being under quarantine. The question on subjective experience of stress was reported on a 7-point Likert scale, time spent virtually socializing, and perception of being under quarantine was a binary “yes/no” response. All questions within the Short Version of the survey were optional and participants were asked to respond to any that they were able to, given their time and energy on the day it was received.

The Full Version of the survey included all questions from the Short Version, as well as measures of mood using the Positive and Negative Affect Schedule (48), subjective experience of worry related to COVID-19, subjective perception of social isolation, and symptoms of depression using a modified version of the Patient Health Questionnaire-9 [PHQ-9; (49)] that omitted the question assessing suicidality. The questions on perception of social isolation and COVID-related worry questions (assessing domains of individual health, family/friend health, community health, national health, financial impact) were reported on 7-point Likert scales. To assess overall worry we created a Worry Composite by summing the responses to all worry questions. The PANAS metrics of positive and negative affect were scored as recommended. The eight remaining PHQ-9 questions were summed as a modified depression score (referred to as “depression” in the results). Most questions within the Full Version were required in order for the form to be submitted, but participation was always optional each day it was received.

Participants were instructed not to try to make up surveys on days that they missed. A view of the Full Version survey questions (that also contains all questions of the Short Version) can be found here: https://bit.ly/BC-FullVersion.

### Study Design

**Figure 1:**
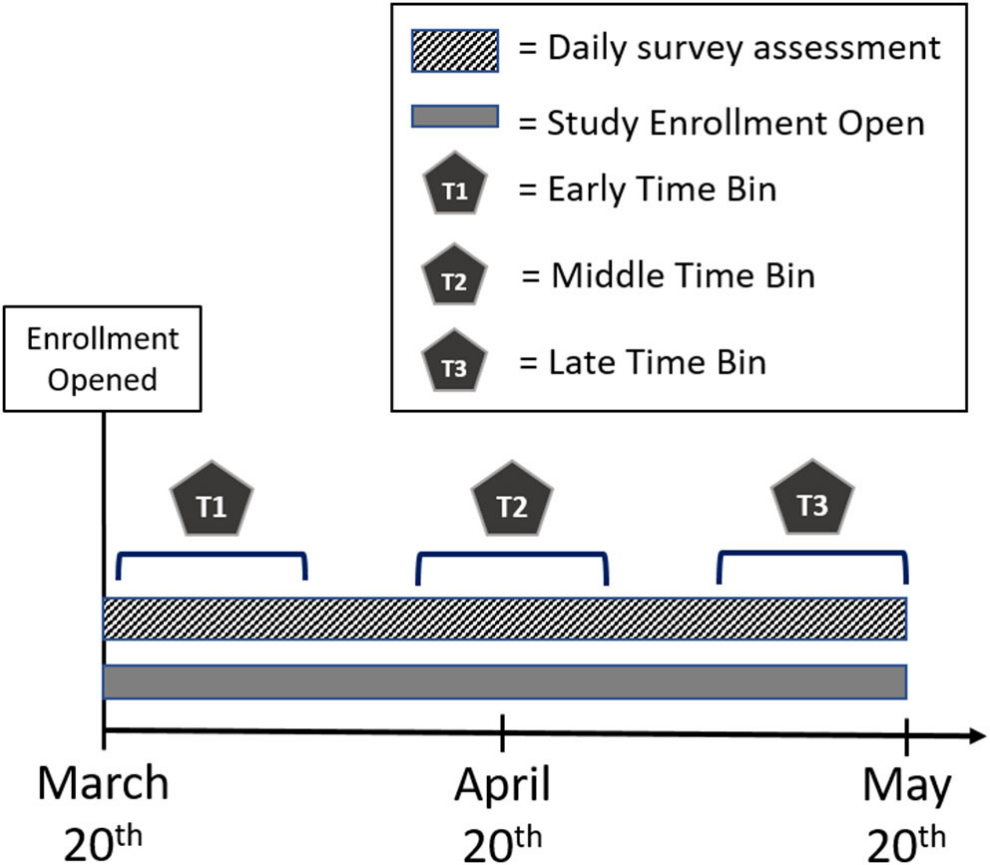
Schematic of study timeline. Enrollment for study began on March 20, 2020. Daily surveys were collected from March 21, 2020 to May 20, 2020. For analyses, we separated the timeline into three separate time bins. Early Time bin (T1) = March 21st–April 3rd; Middle Time bin (T2) = April 14th–April 27th; Late Time bin = May 7th–May 20th.

Data collection procedures are discussed in detail in our pre-registration (https://osf.io/kg6bu). Briefly, enrollment in this study opened on March 20, 2020. After participants completed the online consent form, they received the Demographic Survey. After completion of the demographic survey, they were then enrolled to receive the Daily Surveys for the duration of the assessment period. Participants received either the Short or Full Version of the daily survey each day of the assessment period (until May 20) following their enrollment. To establish a baseline of mental wellbeing, participants received the Full Survey for the first three days following completion of the demographics. The Full Survey was then sent randomly 2 days/week, with the Short Survey sent the remaining 5 days/week. As such, the questions of subjective experience of stress, time spent virtually socializing, and perception of being under quarantine were administered every day of the assessment period, while collection of PANAS positive scale, PANAS negative scale, COVID-related worry questions, subjective social isolation, and the modified PHQ-9 scale occurred twice a week. Although this study is part of a larger study that includes additional planned follow-up assessments, the daily survey data collection ended on May 20, 2020 for all participants. See Figure 1 for a schematic of the study timeline.

### Data Analysis

As described in our pre-registration (https://osf.io/kg6bu), to test our hypotheses, we averaged across all responses for each participant in each time bin (early: March 21st–April 3rd; middle; April 14th–April 27th; late: May 7th–May 20th) for each dependent variable. These time bins were used to create periods of equal duration that were equidistant apart throughout the daily assessment period. The averaged variables were analyzed with linear mixed models with a random intercept for the subject. Each dependent variable of interest was analyzed in a separate model with SGM status and time bin as categorical predictors (Model 1). Average responses to self-reported social isolation and time spent virtual socializing within each time bin were then calculated and added to the models to determine the impact of perceived social isolation (Model 2) and virtual socialization (Model 3) on the relationship between SGM status and time for each metric of mental well-being. For Model 4, responses to each dependent variable of interest were analyzed separately across days that participants reported being under quarantine and days that they were not (ignoring time bin), making quarantine and SGM status categorical predictors for the model. Further, time spent virtually socializing was added to the model to determine the differential impact of socializing virtually on our metrics of mental well-being for participants that reported being under quarantine and those that were not.

Because there was a notable difference in the distribution of age across the SGM and non-SGM groups, all models included age as a covariate. This is particularly important in light of previous analyses on this dataset that determined strong effects of age on most of the reported dependent variables (see https://osf.io/tb4qv). Main effects of age are reported here, but discussed elsewhere (Cunningham et al., in revision). Further, for all models using virtual socialization, the amount of time that participants spent virtually socializing was log (base 2) transformed before being entered into regression models. This reflects the expectation that this variable is more likely to have a logarithmic than linear relationship to mood outcomes—i.e., there will be a benefit of socializing and contact with people, but there will be diminishing returns to the benefit of this variable as the amount increases. This also reduces issues with skew and outliers. Log transformation reduced skew from 4.00 to −0.05 and kurtosis from 30.41 to −0.94. Analyses were conducted in R. Mixed models were conducted with the lme4 (50), lmerTest (51), and afex packages. Jamovi (jamovi.org) and the GAMLj module in jamovi were used to make figures. The data and code used for this analysis is publicly available on Open Science Framework (OSF): https://osf.io/ur27h/.

## Results

Supplementary Table 1 shows correlations among all examined variables.

### Model 1: Effects of SGM and Time

**Figure 2:**
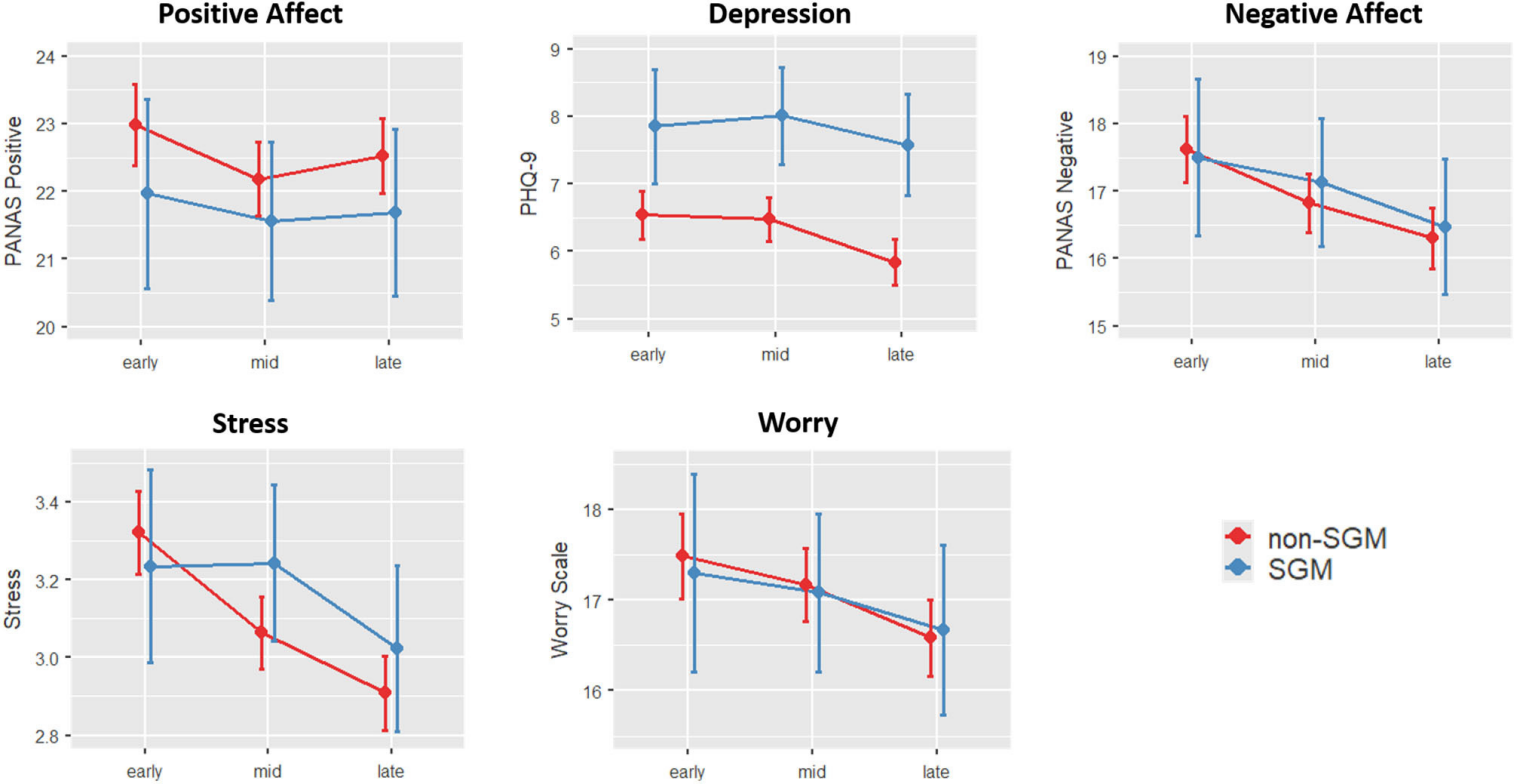
Changes in mood and psychiatric indicators across time and groups (Model 1). Error bars show 95% confidence intervals. Depression was consistently elevated across all three time points for SGM compared to non-SGM. Stress, on the other hand, was differentially affected early on (*p* = 0.028), such that non-SGM reported decreased stress from early to mid, and SGM didn't report a reduction in stress until the late time point.

Coefficients and inferential statistics for the SGM × Time model are shown for all DVs in Table 2. These outcomes are also visualized in Figure 2. To briefly summarize, there was a main effect of Time on negative affect, depression, stress, and worry such that each of these metrics decreased over the course of the three assessment windows (all *p*'s <0.016). There was also a main effect of SGM on depression (*p* < 0.001), but the SGM and non-SGM cohorts did not differ on reports of affect, stress, or worry. There were no significant interactions between SGM status and time across all three assessment windows. For subjective experience of stress, however, there was a trend toward an overall SGM × Time interaction (*p* = 0.089) which became significant when focusing on comparisons between the early and middle time bin (*p* = 0.028). While non-SGM participants showed a reduction in stress between the early and middle time bin, SGM participants showed no such stress reduction; SGM participants did, however, show a reduction in stress between the middle and late time bins, such that by the late time bin, there were no group differences in stress (*p* = 0.111).

**Table 2:** Model 1 regression results.

	**PANAS positive**	**PANAS negative**	**mPHQ-9**	**Stress**	**Worry Composite**
**Intercept**	22.15 [21.55, 22.75]	16.97 [16.50, 17.45]	7.05 [6.68, 7.42]	3.13 [3.03, 3.23]	17.05 [16.61, 17.48]
**Age**	**F(1.00, 961.82)** **=** **177.81,** ***p*** **<** **0.001**	**F(1.00, 936.84)** **=** **18.01,** ***p*** **<** **0.001**	**F(1.00, 949.09)** **=** **31.48,** ***p*** **<** **0.001**	**F(1.00, 974.30)** **=** **36.24,** ***p*** **<** **0.001**	**F(1.00, 957.09)** **=** **8.59,** ***p*** **=** **0.003**
Age	**0.19 [0.16, 0.22],** ***p*** **<** **0.001**	**−0.05 [−0.07**, **−0.03],** ***p*** **<** **0.001**	**−0.05 [−0.07**, **−0.03],** ***p*** **<** **0.001**	**−0.01 [−0.02**, **−0.01],** ***p*** **<** **0.001**	**−0.03 [−0.05**, **−0.01],** ***p*** **=** **0.003**
**SGM_status**	F(1.00, 1,018.84) = 1.81, *p* = 0.179	F(1.00, 1,004.33) = 0.06, *p* = 0.811	**F(1.00, 999.69)** **=** **16.42,** ***p*** **<** **0.001**	F(1.00, 1,058.73) = 0.44, *p* = 0.508	F(1.00, 1,035.16) = 0.02, *p* = 0.889
SGM - non-SGM	−0.83 [−2.03, 0.38], *p* = 0.179	0.12 [−0.84, 1.08], *p* = 0.811	**1.53 [0.79, 2.28],** ***p*** **<** **0.001**	0.07 [−0.14, 0.27], *p* = 0.508	−0.06 [−0.94, 0.81], *p* = 0.889
**Time**	F(2.00, 948.97) = 1.78, *p* = 0.169	**F(2.00, 954.57)** **=** **8.44,** ***p*** **<** **0.001**	**F(2.00, 916.90)** **=** **6.96,** ***p*** **=** **0.001**	**F(2.00, 1,060.91)** **=** **14.04,** ***p*** **<** **0.001**	**F(2.00, 1,008.26)** **=** **4.15,** ***p*** **=** **0.016**
Mid - early	−0.60 [−1.24, 0.03], *p* = 0.063	−0.58 [−1.14, −0.03], *p* = 0.039	0.05 [−0.32, 0.42], *p* = 0.798	**−0.12 [−0.24**, **−0.01],** ***p*** **=** **0.039**	−0.27 [−0.81, 0.28], *p* = 0.340
Late - early	−0.36 [−1.03, 0.30], *p* = 0.286	**−1.18 [−1.75**, **−0.60],** ***p*** **<** **0.001**	**−0.49 [−0.87**, **−0.10],** ***p*** **=** **0.013**	**−0.31 [−0.44**, **−0.19],** ***p*** **<** **0.001**	**−0.76 [−1.33**, **−0.19],** ***p*** **=** **0.009**
**SGM** **×** **Time**	F(2.00, 948.95) = 0.20, *p* = 0.816	F(2.00, 954.54) = 0.29, *p* = 0.745	F(2.00, 916.88) = 0.62, *p* = 0.538	F(2.00, 1,060.81) = 2.42, *p* = 0.089	F(2.00, 1,008.24) = 0.13, *p* = 0.875
(SGM - non-SGM) × (mid –early)	0.39 [−0.88, 1.66], *p* = 0.551	0.43 [−0.67, 1.53], *p* = 0.445	0.22 [−0.52, 0.96], *p* = 0.559	**0.27 [0.03, 0.50],** ***p*** **=** **0.028**	0.10 [−0.99, 1.19], *p* = 0.858
(SGM - non-SGM) × (late –early)	0.17 [−1.16, 1.50], *p* = 0.798	0.28 [−0.87, 1.44], *p* = 0.630	0.43 [−0.34, 1.20], *p* = 0.277	0.20 [−0.05, 0.45], *p* = 0.111	0.28 [−0.86, 1.41], *p* = 0.636

*Coefficients and 95% confidence interval of the coefficient for each effect for each dependent variable. All models included a main effect of age to control for the contribution of age effects. Confidence intervals calculated from the log likelihood ratio test. P-values were calculated from the t-distribution with a Satterthwaite approximation for the degrees of freedom. Coefficients that were significantly different form 0 at α = 0.05 are in bold. mPHQ-9, modified PHQ-9 with all questions except suicidality*.

**Figure 3:**
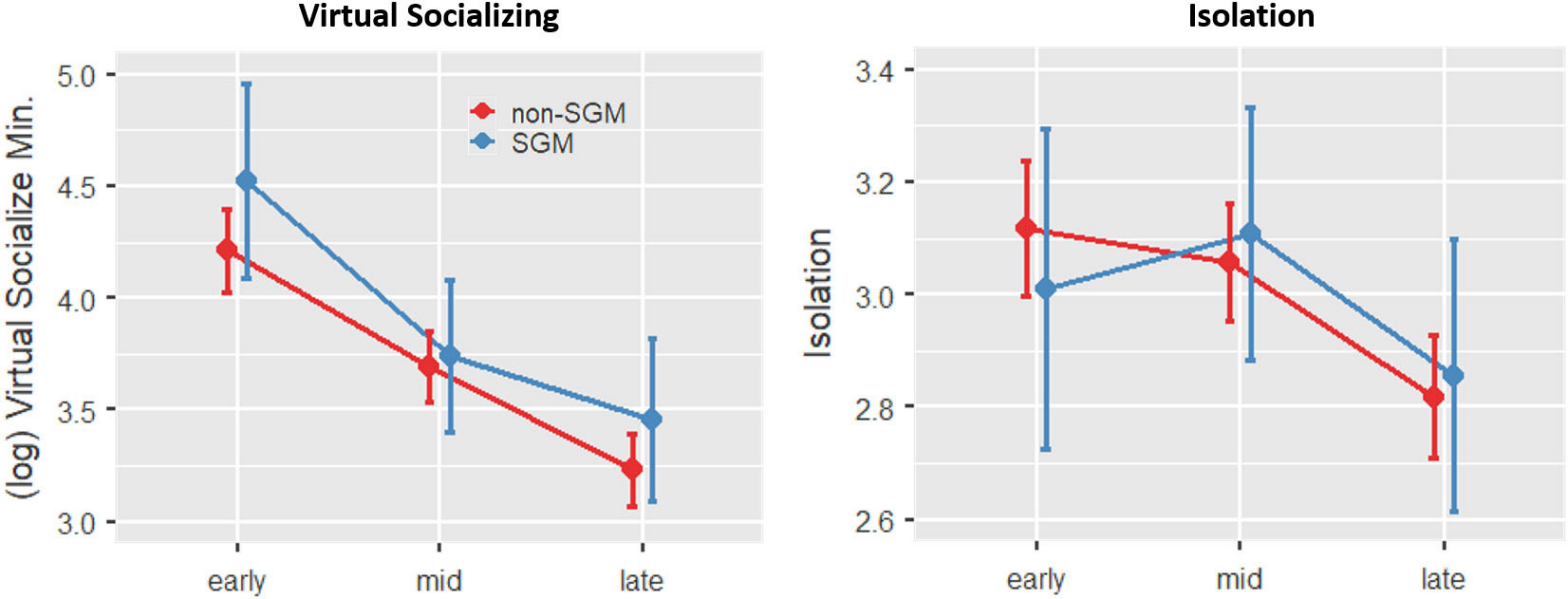
Changes in social engagement and reported feelings of isolation across time and groups (Models 2 and 3). Error bars show 95% confidence intervals. SGM and non-SGM reported similar patterns of progressively decreasing engagement in virtual socializing across the three time points, corresponding to reduced feelings of isolation specifically at the final time point.

When examining the measures of social interaction and isolation as dependent variables to first understand how these variables changed across groups and time, a main effect of Time was observed for both variables (*p*'s <0.001), with both variables generally decreasing over time. There were no effects of SGM status or SGM × Time interaction (Figure 3).

### Model 2: Effects of SGM, Time, and Social Isolation

Coefficients and inferential statistics for the SGM × Time × Social Isolation model are shown in Table 3 and visualized in Supplementary Figure 1. Across all participants, there was a main effect of social isolation such that greater perception of being socially isolated was associated with decreased PANAS positive scale scores and increased PANAS negative, depression, stress, and worry (all *p* < 0.001). There were no significant SGM × Isolation or three-way SGM × Time × Social Isolation interactions for any of the dependent variables of interest. There was a significant Time × Isolation interaction on negative affect and depression, such that the effect of social isolation on these variables was greater in the earlier time bins compared to the late time bin (May 7 - May 20).

**Table 3:** Model 2 regression results.

	**PANAS positive**	**PANAS negative**	**mPHQ9**	**Stress**	**Worry Composite**
**Intercept**	22.28 [21.70, 22.86]	16.87 [16.42, 17.31]	6.93 [6.59, 7.26]	3.08 [3.00, 3.16]	16.95 [16.55, 17.35]
**Age**	**F(1.00, 960.08)** **=** **180.14,** ***p*** **<** **0.001**	**F(1.00, 907.78)** **=** **15.50,** ***p*** **<** **0.001**	**F(1.00, 911.05)** **=** **31.49,** ***p*** **<** **0.001**	**F(1.00, 883.60)** **=** **44.96,** ***p*** **<** **0.001**	**F(1.00, 944.31)** **=** **6.37,** ***p*** **=** **0.012**
Age	**0.19 [0.16, 0.21],** ***p*** **<** **0.001**	**−0.04 [−0.06**, **−0.02],** ***p*** **<** **0.001**	**−0.04 [−0.06**, **−0.03],** ***p*** **<** **0.001**	**−0.01 [−0.02**, **−0.01],** ***p*** **<** **0.001**	**−0.02 [−0.04**, **−0.01],** ***p*** **=** **0.012**
**SGM**	F(1.00, 1,021.44) = 1.86, *p* = 0.172	F(1.00, 985.49) = 0.14, *p* = 0.704	**F(1.00, 973.71)** **=** **19.42,** ***p*** **<** **0.001**	F(1.00, 976.66) = 0.24, *p* = 0.628	F(1.00, 1,027.23) = 0.01, *p* = 0.921
SGM - non-SGM	−0.81 [−1.97, 0.35], *p* = 0.172	0.17 [−0.72, 1.06], *p* = 0.704	**1.51 [0.84, 2.18],** ***p*** **<** **0.001**	0.04 [−0.12, 0.20], *p* = 0.628	−0.04 [−0.85, 0.76], *p* = 0.921
**Time**	F(2.00, 955.47) = 2.20, *p* = 0.112	**F(2.00, 955.74)** **=** **6.00,** ***p*** **=** **0.003**	F(2.00, 909.01) = 2.52, *p* = 0.081	**F(2.00, 984.15)** **=** **5.90,** ***p*** **=** **0.003**	F(2.00, 1,011.52) = 1.19, *p* = 0.306
Mid - early	−0.61 [−1.24, 0.01], *p* = 0.054	**−0.62 [−1.17**, **−0.08],** ***p*** **=** **0.025**	0.08 [−0.28, 0.44], *p* = 0.658	−0.11 [−0.22, 0.00], *p* = 0.058	−0.26 [−0.78, 0.25], *p* = 0.312
Late - early	−0.65 [−1.30, 0.01], *p* = 0.054	**−1.01 [−1.58**, **−0.44],** ***p*** **=** **0.001**	−0.25 [−0.63, 0.13], *p* = 0.203	**−0.20 [−0.32**, **−0.09],** ***p*** **=** **0.001**	−0.42 [−0.96, 0.11], *p* = 0.124
**Isolation**	**F(1.00, 1,783.82)** **=** **60.61,** ***p*** **<** **0.001**	**F(1.00, 1,794.19)** **=** **72.41,** ***p*** **<** **0.001**	**F(1.00, 1,784.75)** **=** **124.45,** ***p*** **<** **0.001**	**F(1.00, 1,756.30)** **=** **296.19,** ***p*** **<** **0.001**	**F(1.00, 1,785.11)** **=** **112.60,** ***p*** **<** **0.001**
Isolation	**−1.23 [−1.53**, **−0.92],** ***p*** **<** **0.001**	**1.10 [0.85, 1.35],** ***p*** **<** **0.001**	**1.02 [0.84, 1.20],** ***p*** **<** **0.001**	**0.43 [0.38, 0.48],** ***p*** **<** **0.001**	**1.26 [1.03, 1.50],** ***p*** **<** **0.001**
**SGM** **×** **Time**	F(2.00, 955.45) = 0.44, *p* = 0.643	F(2.00, 955.72) = 0.15, *p* = 0.863	F(2.00, 908.99) = 0.52, *p* = 0.596	F(2.00, 984.15) = 1.26, *p* = 0.284	F(2.00, 1,011.51) = 0.02, *p* = 0.977
(SGM - non-SGM) × (mid - early)	0.57 [−0.67, 1.81], *p* = 0.372	0.19 [−0.90, 1.28], *p* = 0.735	0.08 [−0.65, 0.80], *p* = 0.836	0.18 [−0.04, 0.40], *p* = 0.113	−0.08 [−1.11, 0.94], *p* = 0.875
(SGM - non-SGM) × (late - early)	0.28 [−1.03, 1.58], *p* = 0.681	−0.04 [−1.19, 1.10], *p* = 0.942	0.34 [−0.43, 1.10], *p* = 0.390	0.13 [−0.11, 0.36], *p* = 0.284	−0.00 [−1.08, 1.08], *p* = 0.998
**SGM** ****×**** **Isolation**	F(1.00, 1,783.92) = 0.19, *p* = 0.666	F(1.00, 1,794.13) = 1.33, *p* = 0.248	F(1.00, 1,784.86) = 0.90, *p* = 0.343	F(1.00, 1,756.14) = 1.49, *p* = 0.223	F(1.00, 1,785.02) = 2.20, *p* = 0.138
(SGM - non-SGM) × isolation	−0.14 [−0.75, 0.48], *p* = 0.666	−0.30 [−0.80, 0.21], *p* = 0.248	0.17 [−0.18, 0.53], *p* = 0.343	−0.06 [−0.16, 0.04], *p* = 0.223	−0.35 [−0.82, 0.11], *p* = 0.138
**Time** **×** **Isolation**	F(2.00, 992.54) = 1.21, *p* = 0.298	**F(2.00, 1,002.91)** **=** **5.23,** ***p*** **=** **0.005**	**F(2.00, 946.81)** **=** **4.71,** ***p*** **=** **0.009**	F(2.00, 1,040.86) = 2.59, *p* = 0.076	F(2.00, 1,061.65) = 1.25, *p* = 0.286
(Mid - early) × isolation	−0.24 [−0.73, 0.25], *p* = 0.337	−0.06 [−0.49, 0.36], *p* = 0.773	0.12 [−0.16, 0.41], *p* = 0.395	**0.09 [0.01, 0.18],** ***p*** **=** **0.035**	0.26 [−0.14, 0.66], *p* = 0.204
(Late - early) × isolation	0.03 [−0.48, 0.53], *p* = 0.922	**−0.53 [−0.97**, **−0.09],** ***p*** **=** **0.018**	−0.20 [−0.49, 0.09], *p* = 0.186	**0.10 [0.01, 0.19],** ***p*** **=** **0.033**	0.33 [−0.08, 0.74], *p* = 0.115
**SGM** **×** **Time** **×** **Isolation**	F(2.00, 992.69) = 0.02, *p* = 0.981	F(2.00, 1,003.09) = 1.12, *p* = 0.328	F(2.00, 946.96) = 0.82, *p* = 0.441	F(2.00, 1,041.03) = 1.01, *p* = 0.364	F(2.00, 1,061.83) = 0.10, *p* = 0.909
(SGM - non-SGM) × (mid - early) × isolation	0.05 [−0.94, 1.03], *p* = 0.928	−0.31 [−1.17, 0.55], *p* = 0.478	−0.31 [−0.88, 0.26], *p* = 0.290	0.00 [−0.17, 0.17], *p* = 0.993	−0.08 [−0.89, 0.72], *p* = 0.840
(SGM - non-SGM) × (late - early) × isolation	−0.03 [−1.03, 0.98], *p* = 0.961	−0.63 [−1.51, 0.24], *p* = 0.160	−0.38 [−0.96, 0.20], *p* = 0.204	−0.09 [−0.26, 0.09], *p* = 0.342	−0.17 [−1.00, 0.65], *p* = 0.682

*Coefficients and 95% confidence interval of the coefficient for each effect for each dependent variable. All models included a main effect of age to control for the contribution of age effects. Confidence intervals calculated from the log likelihood ratio test. P-values were calculated from the t-distribution with a Satterthwaite approximation for the degrees of freedom. Coefficients that were significantly different form 0 at α = 0.05 are in bold. mPHQ-9, modified PHQ-9 with all questions except suicidality*.

### Model 3: Effects of SGM, Time, and Virtual Socialization

Coefficients and inferential statistics for the SGM × Time × Virtual Socialization model are shown in Table 4. There was no main effect of virtual socialization on any metric nor any interactions with virtual socialization. This model did, however, reveal a main effect of Time on PANAS negative, stress, depression (all *p* < 0.001) and worry (*p* < 0.05), with the former two measures showing reductions in these negative mental health consequences between the early and the middle time bin, and with all of those measures showing reductions when comparing the early to the late time bin.

**Table 4:** Model 3 regression results.

	**PANAS positive**	**PANAS negative**	**mPHQ9**	**Stress**	**Worry Composite**
**Intercept**	22.14 [21.54, 22.74]	16.98 [16.50, 17.47]	7.04 [6.67, 7.42]	3.14 [3.04, 3.24]	17.07 [16.63, 17.51]
**Age**	**F(1.00, 962.22)** **=** **172.38,** ***p*** **<** **0.001**	**F(1.00, 940.21)** **=** **18.20**, **<** **0.001**	**F(1.00, 953.70)** **=** **31.64,** ***p*** **<** **0.001**	**F(1.00, 979.38)** **=** **36.27,** ***p*** **<** **0.001**	**F(1.00, 960.53)** **=** **8.72,** ***p*** **=** **0.003**
Age	**0.19 [0.16, 0.22],** ***p*** **<** **0.001**	**−0.05 [−0.07**, **−0.03],** ***p*** **<** **0.001**	**−0.05 [−0.07**, **−0.03],** ***p*** **<** **0.001**	**−0.01 [−0.02**, **−0.01],** ***p*** **<** **0.001**	**−0.03 [−0.05**, **−0.01],** ***p*** **=** **0.003**
**SGM**	F(1.00, 1,030.61) = 1.71, *p* = 0.192	F(1.00, 1,019.56) = 0.01, *p* = 0.916	**F(1.00, 1,013.16)** **=** **15.87,** ***p*** **<** **0.001**	F(1.00, 1,085.23) = 0.33, *p* = 0.567	F(1.00, 1,052.54) = 0.01, *p* = 0.909
SGM - non-SGM	–0.80 [–2.01, 0.40], *p* = 0.192	0.05 [–0.91, 1.02], *p* = 0.916	**1.52 [0.77, 2.26],** ***p*** **<** **0.001**	0.06 [–0.15, 0.27], *p* = 0.567	–0.05 [–0.93, 0.83], *p* = 0.909
**Time**	F(2.00, 999.93) = 1.48, *p* = 0.227	**F(2.00, 1,007.10)** **=** **7.33,** ***p*** **=** **0.001**	**F(2.00, 966.44)** **=** **6.61,** ***p*** **=** **0.001**	**F(2.00, 1,094.28)** **=** **12.95,** ***p*** **<** **0.001**	**F(2.00, 1,062.50)** **=** **3.85,** ***p*** **=** **0.022**
Mid – early	–0.54 [–1.22, 0.14], *p* = 0.122	**−0.60 [−1.18**, **−0.01],** ***p*** **=** **0.048**	0.07 [–0.33, 0.46], *p* = 0.744	**−0.14 [−0.26**, **−0.01],** ***p*** **=** **0.031**	–0.32 [–0.90, 0.26], *p* = 0.276
Late – early	–0.23 [–0.96, 0.50], *p* = 0.537	**−1.18 [−1.81**, **−0.55],** ***p*** **<** **0.001**	**−0.48 [−0.90**, **−0.05],** ***p*** **=** **0.027**	**−0.32 [−0.45**, **−0.19],** ***p*** **<** **0.001**	**−0.80 [−1.42**, **−0.19],** ***p*** **=** **0.011**
**Socialize**	F(1.00, 1,725.80) = 3.15, *p* = 0.076	F(1.00, 1,766.52) = 0.02, *p* = 0.889	F(1.00, 1,667.70) = 0.14, *p* = 0.706	F(1.00, 1,873.41) = 0.01, *p* = 0.913	F(1.00, 1,793.29) = 0.06, *p* = 0.808
Socialize	0.18 [–0.02, 0.38], *p* = 0.076	0.01 [–0.16, 0.18], *p* = 0.889	0.02 [–0.10, 0.14], *p* = 0.706	–0.00 [–0.04, 0.03], *p* = 0.913	0.02 [–0.14, 0.18], *p* = 0.808
**SGM** **×** **Time**	F(2.00, 1,000.10) = 0.15, *p* = 0.863	F(2.00, 1,007.28) = 0.53, *p* = 0.590	F(2.00, 966.62) = 0.46, *p* = 0.630	F(2.00, 1,094.28) = 2.51, *p* = 0.082	F(2.00, 1,062.67) = 0.07, *p* = 0.931
(SGM - non-SGM) × (mid –early)	0.16 [–1.20, 1.53], *p* = 0.817	0.60 [–0.57, 1.78], *p* = 0.317	0.26 [–0.53, 1.05], *p* = 0.517	**0.29 [0.04, 0.54],** ***p*** **=** **0.025**	0.05 [–1.11, 1.21], *p* = 0.933
(SGM - non-SGM) **×** (late –early)	–0.12 [–1.58, 1.34], *p* = 0.870	0.37 [–0.88, 1.62], *p* = 0.566	0.41 [–0.43, 1.26], *p* = 0.338	0.22 [–0.05, 0.48], *p* = 0.111	0.20 [–1.03, 1.43], *p* = 0.753
**SGM** **×** **socialize**	F(1.00, 1,727.51) = 0.95, *p* = 0.330	F(1.00, 1,767.74) = 0.23, *p* = 0.629	F(1.00, 1,669.83) = 0.04, *p* = 0.839	F(1.00, 1,874.41) = 0.06, *p* = 0.814	F(1.00, 1,793.76) = 0.03, *p* = 0.852
(SGM - non-SGM) × socialize	–0.20 [–0.60, 0.20], *p* = 0.330	0.08 [–0.25, 0.42], *p* = 0.629	0.02 [–0.21, 0.26], *p* = 0.839	0.01 [–0.06, 0.08], *p* = 0.814	0.03 [–0.29, 0.35], *p* = 0.852
**Time** **×** **socialize**	F(2.00, 1,030.16) = 0.23, *p* = 0.795	F(2.00, 1,046.84) = 0.22, *p* = 0.806	F(2.00, 989.21) = 0.50, *p* = 0.608	F(2.00, 1,153.60) = 0.52, *p* = 0.594	F(2.00, 1,113.69) = 0.73, *p* = 0.484
(Mid - early) × socialize	0.11 [–0.23, 0.45], *p* = 0.532	0.10 [–0.20, 0.39], *p* = 0.517	0.06 [–0.13, 0.26], *p* = 0.529	0.02 [–0.04, 0.08], *p* = 0.465	0.17 [–0.12, 0.46], *p* = 0.257
(Late - early) × socialize	0.05 [–0.30, 0.40], *p* = 0.781	0.08 [–0.22, 0.39], *p* = 0.587	–0.01 [–0.21, 0.20], *p* = 0.948	0.03 [–0.03, 0.10], *p* = 0.308	0.17 [–0.13, 0.47], *p* = 0.269
**SGM** **×** **Time** **×** **socialize**	F(2.00, 1,030.11) = 0.49, *p* = 0.613	F(2.00, 1,046.78) = 2.04, *p* = 0.131	F(2.00, 989.17) = 1.54, *p* = 0.216	F(2.00, 1,153.59) = 0.23, *p* = 0.793	F(2.00, 1,113.62) = 0.61, *p* = 0.542
(SGM - non-SGM) × (mid –early) × socialize	0.31 [–0.38, 0.99], *p* = 0.384	0.02 [–0.57, 0.61], *p* = 0.943	0.15 [–0.25, 0.54], *p* = 0.468	–0.03 [–0.15, 0.10], *p* = 0.668	0.28 [–0.30, 0.86], *p* = 0.348
(SGM - non-SGM) × (late –early) × socialize	0.35 [–0.36, 1.05], *p* = 0.339	–0.41 [–1.02, 0.19], *p* = 0.184	–0.12 [–0.52, 0.29], *p* = 0.579	–0.04 [–0.17, 0.08], *p* = 0.500	0.08 [–0.51, 0.68], *p* = 0.782

*Coefficients and 95% confidence interval of the coefficient for each effect for each dependent variable. All models included a main effect of age to control for the contribution of age effects. Confidence intervals calculated from the log likelihood ratio test. P-values were calculated from the t-distribution with a Satterthwaite approximation for the degrees of freedom. Coefficients that were significantly different form 0 at α = 0.05 are in bold. mPHQ-9, modified PHQ-9 with all questions except suicidality*.

### Model 4: Effects of SGM, Virtual Socialization, and Reported Quarantine

**Figure 4:**
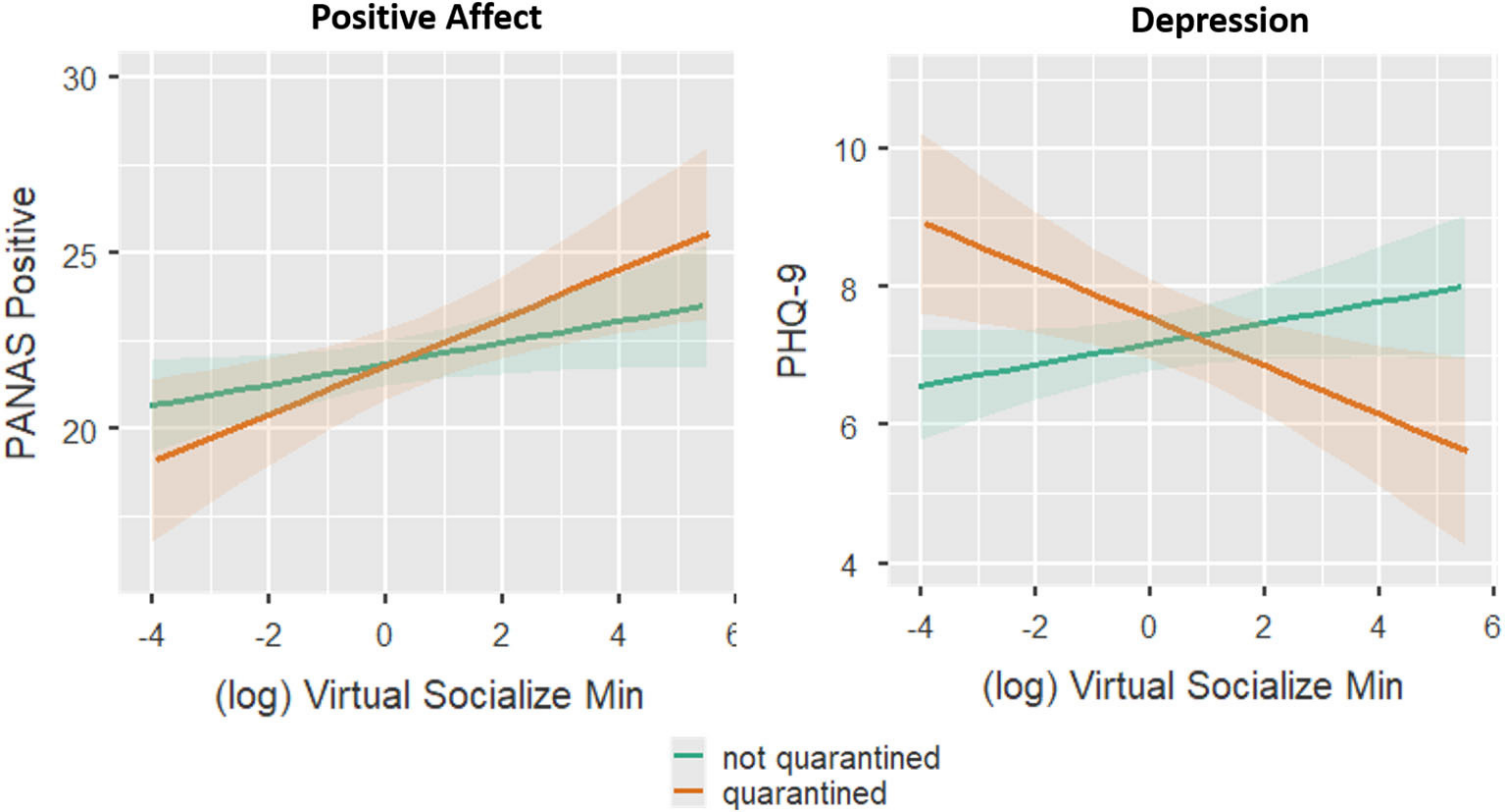
Effects of minutes spent socializing virtually and quarantine on positive affect and depression symptomology (Model 4). Minutes spent socializing virtually is log_2_ transformed (see text) and then mean centered (i.e., 0 represents the mean of the log transformed minutes across all observations). Shading around the lines show the 95% confidence interval.

Coefficients and inferential statistics for the SGM × Virtual Socialization × Quarantine model are shown in Table 5. This model showed a main effect of SGM status on depression (greater depression in those with SGM status). Depression also showed a Quarantine × Virtual Socialization interaction, with Virtual Socialization having a greater reduction on depression in those who reported being quarantined (Figure 4). In this model, a significant effect of Virtual Socialization was revealed on PANAS positive, with increased socialization associated with greater positive affect. This effect may have been significant here but not in Model 3 either because time is removed as a factor in this model or because quarantine is added.

**Table 5:** Model 4 regression results.

	**PANAS positive**	**PANAS negative**	**mPHQ9**	**Stress**	**Worry Composite**
**Intercept**	21.84 [21.19, 22.49]	17.22 [16.69, 17.76]	7.35 [6.95, 7.75]	3.21 [3.10, 3.33]	17.15 [16.67, 17.63]
**Age**	**F(1.00, 991.10)** **=** **156.68,** ***p*** **<** **0.001**	**F(1.00, 979.81)** **=** **19.47,** ***p*** **<** **0.001**	**F(1.00, 990.91)** **=** **31.76,** ***p*** **<** **0.001**	**F(1.00, 999.51)** **=** **35.93,** ***p*** **<** **0.001**	**F(1.00, 985.78)** **=** **8.96,** ***p*** **=** **0.003**
Age	**0.18 [0.15, 0.21],** ***p*** **<** **0.001**	**−0.05 [−0.08**, **−0.03],** ***p*** **<** **0.001**	**−0.05 [−0.07**, **−0.03],** ***p*** **<** **0.001**	**−0.01 [−0.02**, **−0.01],** ***p*** **<** **0.001**	**−0.03 [−0.05**, **−0.01],** ***p*** **=** **0.003**
**SGM**	F(1.00, 1,087.47) = 1.47, *p* = 0.225	F(1.00, 1,086.68) = 0.12, *p* = 0.724	**F(1.00, 1,088.24)** **=** **15.05,** ***p*** **<** **0.001**	F(1.00, 1,132.07) = 2.85, *p* = 0.092	F(1.00, 1,087.11) = 0.11, *p* = 0.739
SGM - non-SGM	–0.82 [–2.13, 0.50], *p* = 0.225	0.19 [–0.88, 1.29], *p* = 0.724	**1.60 [0.80, 2.41],** ***p*** **<** **0.001**	0.19 [–0.03, 0.42], *p* = 0.092	–0.16 [–1.12, 0.79], *p* = 0.739
**Quarantine**	F(1.00, 386.94) = 0.00, *p* = 0.970	F(1.00, 382.66) = 0.94, *p* = 0.333	F(1.00, 272.43) = 1.48, *p* = 0.224	F(1.00, 385.23) = 2.78, *p* = 0.096	F(1.00, 385.84) = 0.10, *p* = 0.758
Quarantined - notquarantined	–0.02 [–1.05, 1.01], *p* = 0.970	0.43 [–0.44, 1.29], *p* = 0.333	0.35 [–0.21, 0.92], *p* = 0.224	0.15 [–0.03, 0.33], *p* = 0.096	0.12 [–0.64, 0.88], *p* = 0.758
**Socialize**	**F(1.00, 834.75)** **=** **11.30,** ***p*** **=** **0.001**	F(1.00, 842.77) = 0.62, *p* = 0.432	F(1.00, 655.73) = 1.34, *p* = 0.247	F(1.00, 793.35) = 0.03, *p* = 0.872	F(1.00, 839.77) = 1.66, *p* = 0.197
(log2) Socialize	**0.49 [0.20, 0.77],** ***p*** **=** **0.001**	0.09 [–0.14, 0.33], *p* = 0.432	–0.10 [–0.26, 0.07], *p* = 0.247	–0.00 [–0.05, 0.04], *p* = 0.872	0.14 [–0.07, 0.35], *p* = 0.197
**SGM** ****×**** **Quarantine**	F(1.00, 388.21) = 0.15, *p* = 0.703	F(1.00, 383.98) = 0.06, *p* = 0.805	F(1.00, 273.19) = 0.14, *p* = 0.707	F(1.00, 386.42) = 1.29, *p* = 0.256	F(1.00, 387.14) = 0.31, *p* = 0.577
(SGM - non-SGM) × (quarantined - notquarantined)	0.40 [–1.65, 2.46], *p* = 0.703	0.22 [–1.54, 1.98], *p* = 0.805	–0.22 [–1.35, 0.92], *p* = 0.707	0.21 [–0.15, 0.56], *p* = 0.256	–0.43 [–1.94, 1.08], *p* = 0.577
**SGM** **×** **Socialize**	F(1.00, 833.48) = 0.08, *p* = 0.776	F(1.00, 841.31) = 0.91, *p* = 0.339	F(1.00, 655.30) = 0.11, *p* = 0.738	F(1.00, 791.94) = 0.24, *p* = 0.625	F(1.00, 838.40) = 1.29, *p* = 0.256
(SGM - non-SGM) × (log2) Socialize	0.08 [–0.49, 0.65], *p* = 0.776	0.23 [–0.24, 0.70], *p* = 0.339	0.06 [–0.28, 0.39], *p* = 0.738	0.02 [–0.07, 0.12], *p* = 0.625	0.24 [–0.17, 0.66], *p* = 0.256
**Quarantine** **×** **Socialize**	F(1.00, 421.89) = 2.18, *p* = 0.140	F(1.00, 420.39) = 2.26, *p* = 0.133	**F(1.00, 290.93)** **=** **12.26,** ***p*** **=** **0.001**	F(1.00, 423.02) = 0.36, *p* = 0.549	F(1.00, 422.27) = 0.17, *p* = 0.678
(Quarantined - notquarantined) × (log2) Socialize	0.38 [–0.12, 0.88], *p* = 0.140	–0.32 [–0.74, 0.10], *p* = 0.133	**−0.50 [−0.77**, **−0.22],** ***p*** **=** **0.001**	–0.03 [–0.11, 0.06], *p* = 0.549	0.08 [–0.29, 0.45], *p* = 0.678
**SGM** **×** **Quarantine** **×** **Socialize**	F(1.00, 421.87) = 2.27, *p* = 0.133	F(1.00, 420.36) = 0.66, *p* = 0.417	F(1.00, 290.93) = 3.07, *p* = 0.081	F(1.00, 422.89) = 0.25, *p* = 0.616	F(1.00, 422.24) = 0.09, *p* = 0.769
(SGM - non-SGM) × (quarantined - notquarantined) × (log2) Socialize	0.78 [–0.23, 1.78], *p* = 0.133	–0.35 [–1.19, 0.49], *p* = 0.417	–0.50 [–1.05, 0.06], *p* = 0.081	0.04 [–0.12, 0.21], *p* = 0.616	–0.11 [–0.85, 0.63], *p* = 0.769

*Coefficients and 95% confidence interval of the coefficient for each effect for each dependent variable. All models included a main effect of age to control for the contribution of age effects. Confidence intervals calculated from the log likelihood ratio test. P-values were calculated from the t-distribution with a Satterthwaite approximation for the degrees of freedom. Coefficients that were significantly different form 0 at α = 0.05 are in bold. mPHQ-9, modified PHQ-9 with all questions except suicidality*.

While there were no main effects of SGM status for any worry variables, exploratory analyses revealed an SGM × Virtual Socialization interaction for financial worry, whereby those with SGM status who socialized more reported more financial worry (see Supplementary Table 2).

### Multiple Comparisons Correction

The multiple models, predictors, and DVs employed in our analysis raise potential multiple comparisons concerns. The main pattern in our results was that there were few significant differences between SGM and cisgender heterosexual participants. That these differences remained non-significant across most DVs and modeling choices with no correction actually gives more confidence in the robustness of this finding (with a strict multiple comparisons correction, non-significant results would be more likely to be due to low power). In addition, the differences that were found, such as greater depression for SGM participants and effects of Time, were generally consistent across models.

Nevertheless, to bolster the interpretation of significant results, we applied the Benjamini and Hochberg (52) false discovery rate (FDR) correction across all *F*-tests in our four main models and five main dependent variables (PANAS positive, PANAS negative, PHQ-9, stress, and the worry scale). Exploratory analyses (e.g., of each separate worry question) were not included in this correction, nor was the effect of age, since this was included simply as a control variable that had already been tested in previous work (Cunningham et al., in revision). This resulted in 126 *p*-values being submitted to the correction algorithm.

The FDR-corrected alpha was ~0.009; in other words, any uncorrected *p* < 0.009 remained significant after FDR correction. As a result, 3 of the 25 effects that were significant in the uncorrected results became non-significant with FDR correction: the Time ^*^ Isolation interaction for PHQ-9 in Model 2, the Time effect for the worry scale in Model 1, and the Time effect for the worry scale in Model 3. Notably, all significant effects that included SGM status remained significant after correction.

## Discussion

The goal of the current study was to investigate changes in mental health outcomes during the initial stages of the US response to the COVID-19 pandemic to determine the extent to which they differed as a function of SGM status. In addition, we sought to explore how perceptions of social isolation and virtual socialization were related to changes in mental health outcomes. Our results suggest few differences in how mental health outcomes changed over time for SGM and cisgender heterosexual persons. Consistent with our initial hypothesis, we found that SGM participants reported greater depression symptoms than cisgender heterosexual individuals across the entirety of the study. However, the rate of change of depression and other mental health outcomes did not differ by SGM status, which was inconsistent with our initial hypotheses. Additionally, though decreased social connectedness and increased time spent engaging in virtual socializing were associated with better mental health, there were no differences by SGM status, again contrary to our hypotheses. These results begin to shed light on potential ways in which the current global pandemic affect mental health among different populations, and must be appreciated with specific reference to the nature of these data. Given that empirical investigations of the mental health impact of COVID-19 are in their infancy, we focus on qualifying our findings, with important directions for future research aimed at holistically understanding mental health vulnerabilities related to the current global pandemic. We believe that these qualifications are particularly noteworthy since these data reflect a non-representative convenience sample of U.S. adults.

When compared with cisgender heterosexual participants, the SGM sample in this study reported significantly younger age. Previous research highlights the particular vulnerabilities faced by older SGM persons to loneliness and social isolation as they age (53–56). Even greater limitations to social connections and increased isolation as a result of the COVID-19 pandemic might impact older SGM adults more so than those represented within our sample. Older SGM individuals are more likely to live alone and lack potential family support systems when compared with their cisgender heterosexual peers (57). Additionally, specific barriers to accessing social support may be potentiated among older SGM individuals (57). Therefore, while these results suggest that the detrimental impact of the pandemic response, and the buffering role that social connectedness and (virtual) activity play, differs little across SGM status, we must qualify this finding by highlighting that these results pertain to *younger-to-middle-aged SGM adults*.

The majority of our SGM sample reported bisexual orientation (~70%), female sex (85%), and cisgender identity (94%). However, despite a robust literature documenting worse health outcomes among bisexual individuals when compared with heterosexual and lesbian/gay individuals (58–62), we found few differences based on SGM status. Nonetheless, bisexual identity is not the driver of mental health disparities *per se*. Instead, previous studies indicate that individuals who report bisexual identity experience greater levels of bi-negativity from both the heterosexual and lesbian/gay communities [e.g., (58–62)]. Minority stress drives mental health disparities. Without measuring experiences/perceptions of bi-negative discrimination or stigma, we are unable to quantify the extent to which such a variable might be related to our findings. For instance, previous research suggests that for bisexual women, the gender of their romantic partner differentially relates to mental health outcomes (58). Approximately half of our SGM participants (55%) reported being involved in romantic relationships. Because we did not collect information on gender identity or sexual orientation of romantic partners, we were unable to explore the potential impact of these variables on our findings. Therefore, it is important to remember the majority bisexual, cisgender representation of our SGM sample when interpreting these results as they likely do not reflect the impact of the social support related to the COVID-19 pandemic across all SGM subgroups.

The majority of our sample reported cisgender identity, and so it is important to note that these findings may be better considered to represent differences between groups as a function of sexual orientation. We utilized an overarching SGM group to maintain fidelity with our preregistered analysis plan. Empirical evidence documents worse psychiatric outcomes for transgender and gender nonconforming individuals when compared with their cisgender counterparts (63–65). It is imperative that future research with greater representation of non-cisgender participants be conducted. Finally, our sample was well educated (>80% with at least a college degree), employed (79%), and mostly non-Hispanic (93%) and white (81%). The epidemiology of psychopathology is stratified according to sexual orientation and race/ethnicity, wherein Hispanic and Black LGB persons experience unalike prevalence of psychiatric disorders when compared with their non-Hispanic white counterparts (19). Socioeconomic status is also inversely associated with mental health outcomes [e.g., (66)]. When considered together, these sample characteristics help us qualify to whom our findings apply. Bearing these qualifications in mind, our results demonstrate that (1) SGM individuals, even those who might belong to social groups with relatively greater psychosocial privilege, nonetheless report worse mental health symptoms when compared with cisgender heterosexual individuals; (2) across the initial phase of the U.S. pandemic response, participants—regardless of sexual orientation and gender identity—experienced decrements in their reported mental health; and (3) social connectedness and maintaining social activities, even in virtual formats, can help buffer against the negative mental health in the face repercussions of the global COVID-9 pandemic.

## Limitations

There are some notable limitations to our study here. First, the data collected here is likely just a snapshot of the impact of the “first wave” of the COVID-19 pandemic in the United States. Even with the start of our data collection beginning in mid-March as cases in the US were initially on the rise and stay-at-home orders were being first introduced, as our measures largely showed improvement over time, it is likely that the uncertainty surrounding the spread of the disease and the severity of its health impact were already taking a substantial toll on mental health and well-being at the start of assessment [for further details see (Cunningham et al., in revision)]. As the pandemic continues to persist and have social and economic impacts, it is possible that the sustained impacts of COVID-19 may begin to again deteriorate mental well-being, and that the chronicity of these stressors may differentially impact different groups. Further, the lack of pre-COVID data limits our capacity to determine if the reported effects would have been the same regardless of the pandemic. We recognize that the current report approaches our question from a biomedical framework, focusing on psychological constructs abstracted from an individual's lived experience and societal factors that may have contributed to them. Our metrics likely do not capture the full extent of potential mental health disparities SGM individuals may be experiencing during this pandemic, nor do we intend to marginalize such experiences. Our use of a binary “yes/no” response to inquire about whether participants were under quarantine at the time of the assessment. Participants reported a number of different interpretations of this question (e.g., medically ordered quarantine vs. extreme social distancing). As such, this question best describes participants' *perception* of whether or not they were “under quarantine.” Further work should explore the differential impact of these different types of isolation and more specifically separate individuals that remained socially active and participants that were socially isolated, whether it was self-imposed or not.

Both sexual and gender minority populations evidence higher prevalence of various forms of psychosocial dysfunction when compared with cisgender heterosexual populations. Given the emerging nature of this literature specifically related to the potential impact of the COVID-19 pandemic on mental health, we felt it important to include gender minority persons within our analyses to avoid erasure of this marginalized population. It is noteworthy that due to the particularly minor representation of gender minority individuals within these data (*n* = 8 transgender participants) we were (1) unable to stratify our analyses by sexual vs. gender minority status and (2) advise caution in the extent to which these conclusions pertain specifically to gender minority persons. Though both sexual and gender minority individuals face psychiatric disparities of a similar nature, grounded in minority stress processes related to their marginalized identities, we reiterate our belief that future studies that better represent the diversity within SGM populations must be pursued to more accurately profile the ways in which the global pandemic might differentially impact already marginalized populations. While we have discussed how the idiosyncratic characteristics of this sample are likely to impact the generalizability of our results, it is important to highlight that these data represent a convenience sample that was limited to individuals that we could reasonably reach using online recruitment techniques. While this work begins to shed light on the potential impact of the COVID-19 pandemic, we want to underscore the importance of replication and expansion of these findings using more representative data.

## Conclusion

The current global COVID-19 pandemic represents an unprecedented period of distress with substantial upheaval to individual's regular lives. Health responses designed to contain the spread of the disease might compromise mental well-being for individuals, with potentially disproportionate impact on marginalized populations who already experience increased psychiatric prevalence. The findings of the current study reveal the buffering effect of social support on preserving individuals' mental health. Although SGM individuals report greater symptoms of depression when compared with their cisgender heterosexual peers, the change of mental health outcomes over time was independent of SGM status. These findings begin to characterize the important mental health effect of the COVID-19 pandemic, and highlight the importance of increasing research aimed at understanding this effect among less homogeneous samples.

## Data Availability Statement

The original contributions presented in the study are publicly available. This data can be found here: https://osf.io/ur27h/.

## Ethics Statement

The studies involving human participants were reviewed and approved by Boston College Institutional Review Board. The patients/participants provided their written informed consent to participate in this study.

## Author Contributions

TC and EK conceived and designed the study. TC conducted data gathering. TC, EF, and EK devised the analysis plan and performed statistical analyses. CR-S, EF, RB, SK, MG, EK, JP, and TC drafted and revised the manuscript. All authors contributed to the article and approved the submitted version.

## Conflict of Interest

The authors declare that the research was conducted in the absence of any commercial or financial relationships that could be construed as a potential conflict of interest.
