# Supplementary material for: Comparing the Impact of COVID-19-Related Social Distancing on Mood and Psychiatric Indicators in Sexual and Gender Minority (SGM) and Non-SGM Individuals
Source: Front Psychiatry. 2020 Dec 22;11:590318. doi: 10.3389/fpsyt.2020.590318 (PMC7783401; doi:10.3389/fpsyt.2020.590318)
Supplement: Supplementary file 1 [file Data_Sheet_1.pdf]

Comparing the Impact of COVID-19-Related Social Distancing on Mood and Psychiatric Indicators in Sexual and Gender Minority (SGM) and Non-SGM Individuals

Craig Rodriguez-Seijas[1], Eric C. Fields[2, 3], Ryan Bottary[3], Sarah M. Kark[4], Michael R. Goldstein[5,6], Elizabeth A. Kensinger[3], Jessica D. Payne[7], Tony J. Cunningham [3,5,6*]

[1]Department of Psychology, University of Michigan, MI, USA

[2]Department of Psychology, Brandeis University, Waltham, MA, USA

[3]Department of Psychology and Neuroscience, Boston College, Chestnut Hill, MA, USA[†]

[4]Department of Neurobiology and Behavior, Center for the Neurobiology of Learning and Memory, University of California at Irvine, Irvine, California, USA

[5]Department of Psychiatry, Harvard Medical School, Boston, MA, USA

[6]Department of Psychiatry, Beth Israel Deaconess Medical Center, Boston, MA, USA

[7]Department of Psychology, University of Notre Dame, Notre Dame, IN, USA

† Institution where work was conducted.

*** Correspondence:**
Tony J. Cunningham

acunnin4@bidmc.harvard.edu

Supplementary Material

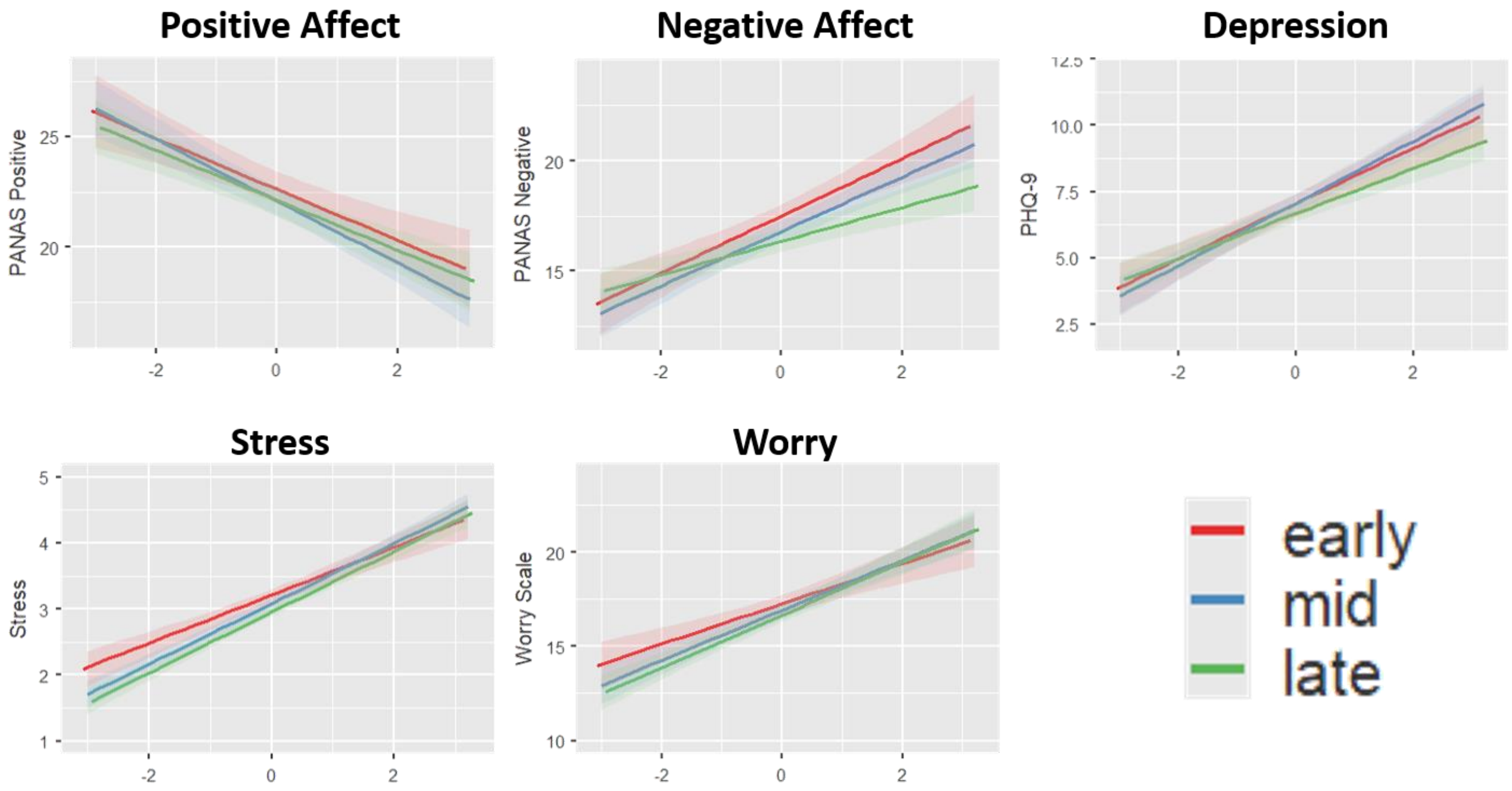

Supplementary Figure 1. Effects of self-reported social isolation and time window on mood and psychiatric symptoms (Model 2). Isolation is mean centered (i.e., 0 represents the mean response across all observations). Shading around the lines show the 95% confidence interval.

Supplementary Table 1: Pearson correlations between all examined variables. Red colors highlight negative correlations and Green highlight positive correlations. Bolded values are significant with alpha = 0.05. mPHQ-9 = modified PHQ-9 with all questions except suicidality

	Age	SGM status	Time (linear)	Time (quadratic)	Isolation	virtual socialize minutes (log2)	PANAS positive	PANAS negative	mPHQ-9	Stress	Worry Composite	Quarantine
Age	—											
SGM status	**-0.18**	—										
Time (linear)	0.02	0.01	—									
Time (quadratic)	0.03	-0.04	**0.14**	—								
Isolation	**-0.06**	0.01	**-0.07**	**-0.08**	—							
(log2) virtual socialize minutes	**0.10**	0.00	**-0.17**	0.01	-0.03	—						
PANAS positive	**0.42**	**-0.11**	-0.02	**0.05**	**-0.27**	**0.16**	—					
PANAS negative	**-0.13**	0.04	**-0.10**	-0.01	**0.38**	0.01	**-0.10**	—				
mPHQ-9	**-0.20**	**0.15**	-0.01	**-0.10**	**0.43**	-0.04	**-0.38**	**0.61**	—			
Stress	**-0.20**	**0.05**	**-0.10**	-0.03	**0.60**	-0.03	**-0.33**	**0.61**	**0.55**	—		
Worry Composite	**-0.10**	0.02	**-0.06**	-0.02	**0.42**	-0.03	**-0.13**	**0.42**	**0.32**	**0.63**	—	
Quarantine	**-0.11**	0.03	0.04	**-0.05**	**0.09**	**0.05**	-0.01	**0.05**	**0.07**	0.04	0.01	—

Supplementary Table 2: Model 4 Results with individual worry questions. Coefficients and 95% confidence interval of the coefficient for each effect for each dependent variable. All models included a main effect of age to control for the contribution of age effects. Confidence intervals calculated from the log likelihood ratio test. P-values were calculated from the t-distribution with a Satterthwaite approximation for the degrees of freedom. Coefficients that were significantly different form 0 at α = 0.05 are in bold.

	**Worry – Personal Health**	**Worry – Family Health**	**Worry- Community Health**	**Worry -National Health**	**Worry - Finances**
**intercept**	2.47 [2.34, 2.60]	3.61 [3.49, 3.72]	3.59 [3.47, 3.70]	4.10 [3.99, 4.21]	3.37 [3.24, 3.51]
**Age**	**F(1.00, 978.42) = 7.15, p = .008**	**F(1.00, 993.73) = 22.33, p < .001**	**F(1.00, 982.61) = 3.93, p = .048**	**F(1.00, 978.95) = 35.67, p < .001**	**F(1.00, 973.36) = 5.72, p = .017**
Age	**0.01 [0.00, 0.01]**	**-0.01 [-0.02, -0.01]**	**-0.01 [-0.01, -0.00]**	**-0.01 [-0.02, -0.01]**	**-0.01 [-0.01, -0.00]**
**SGM**	F(1.00, 1084.62) = 0.09, p = .762	F(1.00, 1085.34) = 1.35, p = .246	F(1.00, 1085.61) = 0.32, p = .569	F(1.00, 1083.59) = 0.65, p = .419	F(1.00, 1084.82) = 0.98, p = .322
SGM - non-SGM	-0.04 [-0.30, 0.22]	-0.14 [-0.38, 0.10]	0.07 [-0.16, 0.30]	0.09 [-0.13, 0.32]	-0.14 [-0.41, 0.13]
**Quarantine**	F(1.00, 437.44) = 0.04, p = .836	F(1.00, 479.66) = 0.15, p = .702	F(1.00, 427.35) = 0.55, p = .460	F(1.00, 461.98) = 0.28, p = .594	F(1.00, 414.87) = 0.42, p = .517
quarantined - not quarantined	0.02 [-0.19, 0.24]	-0.04 [-0.24, 0.16]	0.07 [-0.12, 0.26]	-0.05 [-0.24, 0.14]	0.07 [-0.15, 0.30]
**Socialize**	F(1.00, 904.21) = 2.74, p = .098	**F(1.00, 926.85) = 4.20, p = .041**	F(1.00, 889.63) = 0.90, p = .344	F(1.00, 926.67) = 0.64, p = .423	**F(1.00, 886.42) = 6.52, p = .011**
(log2) Socialize	-0.05 [-0.11, 0.01]	**0.06 [0.00, 0.11]**	0.02 [-0.03, 0.08]	0.02 [-0.03, 0.07]	**0.08 [0.02, 0.14]**
**SGM x Quarantine**	F(1.00, 438.96) = 1.05, p = .307	F(1.00, 481.21) = 0.41, p = .520	F(1.00, 428.81) = 0.29, p = .592	F(1.00, 463.56) = 0.01, p = .904	F(1.00, 416.34) = 0.10, p = .746
(SGM - non-SGM) x (quarantined - not quarantined)	-0.22 [-0.65, 0.20]	-0.13 [-0.52, 0.26]	-0.10 [-0.48, 0.27]	-0.02 [-0.40, 0.35]	0.07 [-0.37, 0.52]
**SGM x Socialize**	F(1.00, 902.59) = 0.74, p = .391	F(1.00, 925.41) = 1.12, p = .290	F(1.00, 888.09) = 0.57, p = .450	F(1.00, 925.05) = 0.17, p = .679	**F(1.00, 884.77) = 7.43, p = .007**
(SGM - non-SGM) x (log2) Socialize	-0.05 [-0.17, 0.06]	0.06 [-0.05, 0.16]	0.04 [-0.06, 0.14]	0.02 [-0.08, 0.12]	**0.17 [0.05, 0.29]**
**Quarantine x Socialize**	F(1.00, 484.21) = 2.45, p = .118	F(1.00, 527.10) = 1.70, p = .192	F(1.00, 471.14) = 0.44, p = .509	F(1.00, 512.36) = 0.58, p = .445	F(1.00, 459.70) = 0.37, p = .544

(quarantined - not quarantined) x (log2) Socialize	-0.08 [-0.19, 0.02]	0.06 [-0.03, 0.16]	0.03 [-0.06, 0.12]	0.04 [-0.06, 0.13]	0.03 [-0.08, 0.14]
**SGM x Quarantine x Socialize**	F(1.00, 484.18) = 3.25, p = .072	F(1.00, 527.07) = 0.02, p = .896	F(1.00, 471.11) = 0.07, p = .796	F(1.00, 512.34) = 0.61, p = .435	F(1.00, 459.67) = 0.82, p = .365
(SGM - non-SGM) x (quarantined - not quarantined) x (log2) Socialize	-0.19 [-0.40, 0.02]	0.01 [-0.18, 0.20]	0.02 [-0.16, 0.21]	-0.07 [-0.26, 0.11]	0.10 [-0.12, 0.32]
